# An Alanine-to-Valine Substitution in the Residue 175 of Zika Virus NS2A Protein Affects Viral RNA Synthesis and Attenuates the Virus In Vivo

**DOI:** 10.3390/v10100547

**Published:** 2018-10-07

**Authors:** Silvia Márquez-Jurado, Aitor Nogales, Ginés Ávila-Pérez, Francisco J. Iborra, Luis Martínez-Sobrido, Fernando Almazán

**Affiliations:** 1 Department of Molecular and Cell Biology, Centro Nacional de Biotecnología (CNB-CSIC), Campus Universidad Autónoma de Madrid, 3 Darwin street, 28049 Madrid, Spain; smarquez@cnb.csic.es (S.M.-J.); fjiborra@cnb.csic.es (F.J.I.); 2 Department of Microbiology and Immunology, University of Rochester Medical Center, 601 Elmwood Avenue, Rochester, NY 14642, USA; aitor_nogales@urmc.rochester.edu (A.N.); Gines_Perez@urmc.rochester.edu (G.Á.P.)

**Keywords:** Zika virus, Full-length cDNA infectious clones, Bacterial artificial chromosome, NS2A protein

## Abstract

The recent outbreaks of Zika virus (ZIKV), its association with Guillain–Barré syndrome and fetal abnormalities, and the lack of approved vaccines and antivirals, highlight the importance of developing countermeasures to combat ZIKV disease. In this respect, infectious clones constitute excellent tools to accomplish these goals. However, flavivirus infectious clones are often difficult to work with due to the toxicity of some flavivirus sequences in bacteria. To bypass this problem, several alternative approaches have been applied for the generation of ZIKV clones including, among others, in vitro ligation, insertions of introns and using infectious subgenomic amplicons. Here, we report a simple and novel DNA-launched approach based on the use of a bacterial artificial chromosome (BAC) to generate a cDNA clone of Rio Grande do Norte Natal ZIKV strain. The sequence was identified from the brain tissue of an aborted fetus with microcephaly. The BAC clone was fully stable in bacteria and the infectious virus was efficiently recovered in Vero cells through direct delivery of the cDNA clone. The rescued virus yielded high titers in Vero cells and was pathogenic in a validated mouse model (A129 mice) of ZIKV infection. Furthermore, using this infectious clone we have generated a mutant ZIKV containing a single amino acid substitution (A175V) in the NS2A protein that presented reduced viral RNA synthesis in cell cultures, was highly attenuated in vivo and induced fully protection against a lethal challenge with ZIKV wild-type. This BAC approach provides a stable and reliable reverse genetic system for ZIKV that will help to identify viral determinants of virulence and facilitate the development of vaccine and therapeutic strategies.

## 1. Introduction

Zika virus (ZIKV) is a recently emerged mosquito-borne member of the family *Flaviviridae*, which was declared by the Word Health Organization (WHO) as a global public health emergency on February 2016 [1,2]. Like other flaviviruses, the viral particle is constituted by an inner nucleocapsid composed of the capsid (C) protein associated with the viral genomic RNA (gRNA), surrounded by a lipid bilayer that contains the structural membrane (M) and envelope (E) proteins, which are arranged with icosahedral symmetry on the surface [3]. The viral genome consists in a positive single-stranded RNA molecule of about 10.8 kb that, similarly to cellular mRNAs, contains a cap structure at the 5’ end and a single open reading frame (ORF) flanked by 5’ and 3’ untranslated regions (UTRs). The ORF encodes a large polyprotein of approximately 3423 amino acids, which is co- and post-translationally processed by viral and cellular proteases into three structural proteins (C, pre-membrane (prM) and E) and seven non-structural (NS) proteins (NS1, NS2A, NS2B, NS3, NS4A, NS4B and NS5). The structural proteins are essential components of the virion and are involved in viral entry, fusion, and assembly. The NS proteins are required for viral RNA synthesis, virion assembly and evasion of the host antiviral responses [4,5].

Human illness caused by ZIKV infection was first recognized in Nigeria in 1953 [6] and only a few cases of ZIKV infections across Africa and Asia were reported over 50 years. Historically, ZIKV was considered as a modest public health concern, causing a mild febrile illness with similar symptoms to those of dengue (DENV) and chikungunya (CHIKV) viruses, hampering differential diagnosis [7]. In general, over 80% of ZIKV cases are asymptomatic, while the remaining cases typically exhibit mild fever, maculopapular rash, and joint pain for a period of several days to a week [8]. However, the recent large outbreaks in the Island of Yap in 2007 [9], French Polynesia in 2013 [10], and the massive epidemic that emerge from Brazil in 2015 to rapidly spread throughout South and Central America, the Caribbean, and more recently the United States [1,11,12,13], have changed the historic perspective of ZIKV infection due its association with Guillain–Barré syndrome (a debilitating neuronal disease in adults) and severe congenital abnormalities such us stillbirth, hydrocephaly and microcephaly, which are collectively known as congenital ZIKV syndrome (CZVS) [14,15,16,17,18,19]. Human health concerns posed by ZIKV are further aggravated due to the non-vector-borne transmission of ZIKV. The virus is spread to people primarily through the bite of infected mosquitoes of the genus *Aedes* [20,21], however, at difference to most other flaviviruses, ZIKV can also be transmitted from mother to child during pregnancy or spread through sexual contact, breastfeeding, blood transfusion, and non-human primate bites [1,22,23]. Due to the recent emergence of ZIKV as an important human pathogen, there are not currently approved vaccines or antivirals available to combat ZIKV infection. The only available disease prevention measures consist in protection from mosquito bites, excluding pregnant females from travelling to ZIKV-endemic areas, and practicing safe sex.

The significance of ZIKV in human health, together with the lack of prophylactic and therapeutic interventions to combat ZIKV infection, highlight the importance of developing safe and effective countermeasures to control or prevent ZIKV disease in humans. In this sense, the development of ZIKV reverse genetic systems constitute an essential tool for basic research and development of vaccine and antiviral strategies. Likewise other flaviviruses, construction of ZIKV infectious clones has been hampered due to the toxicity of some flavivirus sequences during its propagation in bacteria using standard high copy number plasmids, which can be attributed to the leaky expression of toxic viral proteins from cryptic bacterial promoters (CBPs) encoded in the viral genome [24,25,26]. Recently, this toxicity problem was overcome using non-traditional approaches based on in vitro ligation of cDNA fragments [27,28], low-copy plasmids [29,30], intron insertion in the toxic region [31,32,33], Gibson assembly method [34], infectious subgenomic amplicons (ISA) [35,36], in silico prediction and mutational silencing of CBPs present in the viral genome [37], and the use of circular polymerase extension reaction (CPER) [38]. All these systems are valuable tools to study viral pathogenesis, vector transmission and for the development of attenuated forms of ZIKV for their implementation as safe vaccines or for the identification of therapeutics.

In the present study, we report a different and efficient ZIKV reverse genetic approach, based on the use of a bacterial artificial chromosome (BAC), that overcomes the toxicity problems and allows the generation of ZIKV cDNA clones on single bacterial plasmid. Following a similar strategy to that used for DENV [39], the full-length cDNA copy of the viral genome of ZIKV Rio Grande do Norte Natal (RGN) was assembled in a BAC under the control of the cytomegalovirus (CMV) immediate-early promoter. This DNA-launched system couples expression of the viral RNA in the nucleus from the CMV promoter with a second amplification step in the cytoplasm driven by the viral polymerase. The recombinant virus rescued from the BAC clone was fully infectious in vitro and in vivo. The ZIKV-RGN infectious clone was further used to evaluate the effect of a single amino acid change (alanine to valine) at residue 175 of the NS2A protein on viral RNA synthesis and pathogenesis in vivo. We found that this unique single amino acid substitution impairs viral RNA synthesis in cell culture and results in viral attenuation in A129 mice. Remarkably, a single dose of the mutant virus was sufficient to induce protection against challenge with the parental wild-type (WT) ZIKV. These results demonstrate the reliability and potential of our BAC approach to study ZIKV biology and to facilitate the development of vaccine and antiviral strategies.

## 2. Materials and Methods

### 2.1. Cell Culture and Virus Infection

Vero (a kidney epithelial cell line from an African green monkey) and A549 (an human adenocarcinomic alveolar epithelial cell line) cells were purchased from the American Type Culture Collection (ATCC, CCL-81) and were grown and maintained at 37 °C and 5% CO_2_ in growth medium, consisting in Dulbecco’s modified Eagle’s medium (DMEM) supplemented with 5% fetal bovine serum (FBS) (HyClone, ThermoFisher Scientific, Madrid, Spain), 2 mM l-glutamine (Sigma-Aldrich, Madrid, Spain), 1% nonessential amino acids (Sigma-Aldrich), 100 U/mL penicillin (Sigma-Aldrich) and 100 µg/mL streptomycin (Sigma-Aldrich).

The recombinant ZIKV-RGN WT (rZIKV-RGN) or NS2A mutant (rZIKV-RGN-mNS2A) viruses were propagated in Vero cells with virus growth medium (DMEM supplemented with 2% FBS, 2 mM l-glutamine, 1% nonessential amino acids, 100 U/mL penicillin and 100 µg/mL streptomycin) at 37 °C and 5% CO_2_. For virus stocks preparation, 80 to 90% confluent monolayers of Vero cells were infected with a multiplicity of infection (MOI) of 0.1 plaque forming units (PFU) per cell in virus growth medium and incubated at 37 °C under 5% CO_2_. After 3–4 days of infection, the tissue culture supernatants were collected, clarified by centrifugation at 6000× *g* for 5 min, and stored in small aliquots at −80 °C.

### 2.2. Plasmids and Bacteria Strains

The BAC plasmid pBeloBAC11 [40], kindly provided by H. Shizuya (California Institute of Technology, Pasadena, CA, USA), was used to assemble the ZIKV-RGN infectious cDNA clone. This plasmid is a synthetic low-copy-number plasmid (one copy per cell) based on the *Escherichia coli* (*E. coli)* F-factor [41] that minimize the toxicity problems in the bacteria of exogenous sequences. *E. coli* DH10B cells (Invitrogen, ThermoFisher Scientific) were used to amplify the BAC plasmids. Electrocompetent DH10B cells (Invitrogen, ThermoFisher Scientific) were transformed by electroporation using a MicroPulser unit (Bio-Rad, Madrid, Spain), according to the manufacturer’s instructions. BAC-based plasmids were isolated and purified using the Large-Construct kit (Qiagen, Hilden, Germany), following the manufacturer’s specifications.

### 2.3. Construction of ZIKV-RGN Infectious cDNA Clone

**Figure 1:**
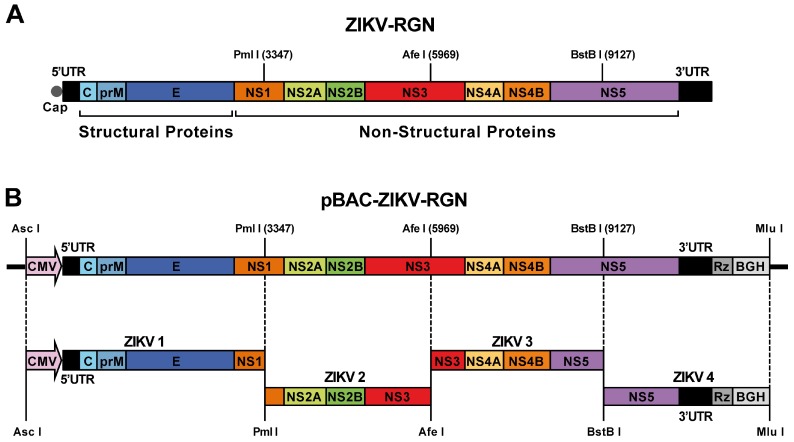
Assembly of a Zika Virus-Rio Grande do Norte Natal (ZIKV-RGN) infectious cDNA clone as a bacterial artificial chromosome (BAC). (**A**) Genetic structure of the ZIKV-RGN strain genome. The coding region from the structural (C, prM and E) and NS (NS1, NS2A, NS2B, NS3, NS4A, NS4B and NS5) proteins are illustrated by colored boxes. Relevant unique restriction sites in the viral genome used for the assembly of the infectious clone and their genomic positions (in brackets) are indicated. 5’ and 3’ UTR: 5’ and 3’ untranslated regions; cap: cap structure. (**B**) Strategy to assemble the ZIKV-RGN infectious cDNA clone. Four overlapping DNA fragments (ZIKV 1 to ZIKV 4, left to right), covering the entire viral genome and flanked by the indicated restriction sites, were generated by chemical synthesis and sequentially cloned into the BAC plasmid pBeloBAC11 to generate the infectious BAC clone pBAC-ZIKV-RGN. The full-length cDNA was assembled under the control of the cytomegalovirus (CMV) immediate-early promoter and flanked at the 3’-end by the hepatitis delta virus (HDV) ribozyme (Rz) followed by the bovine growth hormone (BGH) termination and polyadenylation sequences. Acronyms for coding regions and regulatory elements are as described in panel A.

We have assembled a ZIKV infectious cDNA clone in the BAC plasmid pBeloBAC11, based on the data of the full-length sequence of the ZIKV clinical strain RGN [19] deposited in the GenBanK (accession number KU527068). This strain was selected because the full-length sequence was obtained directly from the virus-infected brain tissue of an aborted fetus with microcephaly in Brazil in 2015 and therefore represents a good candidate to study ZIKV pathogenesis. The first step for the assembly of the full-length cDNA clone was the selection of the restriction sites PmlI, AfeI, and BstBI (genomic positions 3347, 5969 and 9127, respectively), which are unique in the viral genome (Figure 1A). After that, four overlapping DNA fragments covering the entire viral genome (ZIKV 1 to ZIKV 4) and flanked by the appropriate restriction sites, were generated by chemical synthesis (Bio Basic, Inc., Toronto, Canada) (Figure 1B). ZIKV 1 fragment contained the CMV promoter precisely fused to the first 3350 nucleotides of the viral genome flanked at the 5’-end by ApaLI and AscI (absent in the viral genome) sites and at the 3’-end by a multiple-cloning site containing the selected restriction sites (PmlI, AfeI and BstBI) followed by MluI (absent in the viral genome) and BamHI. Fragments ZIKV 2 (flanked by PmlI and AfeI) and ZIKV 3 (flanked by AfeI and BstBI) covered the genomic regions 3346–5972 and 5967–9131, respectively. ZIKV 4 fragment contained the restriction site BstBI, the last 1683 nucleotides of the viral genome, the hepatitis delta virus (HDV) ribozyme, the bovine growth hormone (BGH) termination and polyadenylation sequences, and the MluI restriction site. The infectious clone was assembled into pBeloBAC11 by sequential cloning of these four overlapping DNA fragments. Briefly, fragment ZIKV 1 was digested with ApaLI and BamHI and cloned into pBeloBAC11^−AfeI^ (a pBeloBAC11 without the AfeI restriction site) digested with the same enzymes, to generate the intermediate plasmid pBAC-ZIKV1. Then, this plasmid was used as the backbone for the sequential cloning of the remaining overlapping DNA fragments (ZIKV 2 to ZIKV 4) into the multicloning site of the intermediate plasmid (contains the restriction sites selected, PmlI, AfeI, BstBI and MluI) to generate the full-length cDNA clone pBAC-ZIKV-RGN (Figure 1B). The genetic integrity of the cDNA clone was verified throughout the assembly process by extensive restriction analysis and sequencing. In all cases, the bacterial strain DH10B (Invitrogen, ThermoFisher Scientific) was used as the *E. coli* host for all the cloning steps and the propagation of the BAC cDNA clone.

### 2.4. Recovery of Infectious Virus from the BAC cDNA Clones

To recover the infectious virus, Vero cells on 6-well plates were grown to 90% confluence in growth medium without antibiotics, and transfected with 4 µg of the BAC cDNA clone using 12 µL of Lipofectamine 2000 (Invitrogen, ThermoFisher Scientific), following the manufacturer’s specifications. After 6 h of incubation at 37 °C, the transfection medium was replaced with fresh growth medium and the cells incubated at 37 °C. Aliquots of the culture supernatants were collected at 24 h intervals for virus titer determination by plaque assay on Vero cells. After five to seven days of transfection, when the cytopathic effect (CPE) was clear, cell culture supernatants were harvested and the recovered virus was cloned by three rounds of plaque purification.

### 2.5. Sequencing of Viral RNA

To determine the complete genome sequence of the rescued viruses, virions from supernatant of infected Vero cells (MOI of 0.01 PFU/cell) were purified through a 20% (*w*/*v*) sucrose cushion. Viral RNA was isolate from the purified virus with the QIAamp viral RNA minikit (Qiagen) following the manufacturer’s instructions and deep-sequenced at the University of Rochester Genomics Research Center using Illumina MiSeq (Illumina, San Diego, CA, USA). Briefly, 0.5 µg of total viral RNA was fragmented by controlled sonication and a DNA library was generated using the NEBNext mRNA library prep master mix set for Illumina (New England Biolabs, Ipswich, MA, USA), according to the manufacturer’s instructions. After analyzing the library for size and quality (Bio-Analyzer; Agilent Technologies, Inc., Santa Clara, CA, USA), deep-sequencing was performed using MiSeq (Illumina) and the raw sequencing reads analyzed using SWARM custom software. The genomic 5’- and 3’-terminal sequences were determined by the rapid amplification of cDNA ends (RACE) using the 5’/3’ RACE second generation kit (Roche, Basilea, Switzerland) with a polyA-tail added to the cDNA prior to the 3’ RACE reaction using polyA polymerase (New England Biolabs), following the manufacturer’s instructions.

To analyze the genetic stability of the recombinant ZIKV harboring the point mutation A175V in the coding region of the NS2A protein (rZIKV-RGN-mNS2A), total RNA was purified from Vero cells infected with viruses from passage 1 (P1) to passage 5 (P5) using the RNeasy minikit (Qiagen), according to the manufacturer’s specifications. Purified RNA (600 ng) was reverse transcribed (RT) with random hexamer primers using the High-Capacity cDNA Transcription kit (Life Technologies, ThermoFisher Scientific), and the cDNA was amplified by PCR with the forward primer ZIKV-3414VS (5’-GAGGAATGGTGCTGCAGG-3’), spanning nucleotides 3414 to 3431 of the viral genome, and the reverse primer ZIKV-4817RS (5’-GCTTGACATCTCCCCAG-3’), complementary to nucleotides 4817 to 4833 of the viral genome. Finally, the amplicons generated covering the region encoding NS2A and NS2B proteins (genomic region 3414–4833) were sequenced by Sanger sequencing (Macrogen Europe, Amsterdam, Netherlands) using specific oligonucleotides.

### 2.6. Virus Titrations

Vero cells seeded into 12-well plates at 80–90% of confluence were infected with 150 µL of serial 10-fold dilutions of the virus in virus growth medium without FBS for 1 h at 37 °C. After viral absorption, the viral inoculum was removed and the cells overlaid with 2 mL of virus growth medium containing 1% DEAE-Dextran (Sigma-Aldrich) and 0.6% Agar Noble (Difco, ThermoFisher Scientific). After 3–4 days of incubation at 37 °C under 5% CO_2_, the cells were fixed with 4% formaldehyde for 1 h at room temperature, the overlaid removed, and the viral plaques visualized by staining with 0.1% crystal violet in 20% methanol or by immunostaining with 1 µg/mL of the pan-flavivirus E protein monoclonal antibody (mAb) 4G2 (BEI Resources; NR-50327) using the Vectastain ABC kit (Vector Laboratories Inc., Burlingame, CA, USA). Visible plaques were counted and virus titers were calculated as PFU/mL.

### 2.7. Virus Growth Kinetics

Vero and A549 cells seeded into 24-well plates at 90% of confluence were infected with the indicated viruses diluted in virus growth medium without FBS at the specified MOIs. After 1 h of absorption at 37 °C in 5% CO_2_, the virus inoculum was removed, the cell monolayers washed twice with PBS, and 0.5 mL of fresh virus growth medium was added to each well. Cells were incubated at 37 °C under 5% CO_2_ and at selected time points, aliquots of tissue culture supernatants were collected and virus titers determined by plaque assay in Vero cells as described above.

### 2.8. Analysis of Viral RNA Synthesis

Viral RNA synthesis was evaluated by quantitative RT-PCR (RT-qPCR). Total intracellular RNA from uninfected or infected Vero cells was purified using the RNeasy minikit (Qiagen) and total cDNA was synthetized from 100 ng of purified RNA using random hexamer primers and the High-Capacity cDNA Transcription kit (Life Technologies, ThermoFisher Scientific), following the manufacturer’s specifications. Using this cDNA, the level of viral RNA was further quantified by qPCR using a custom TaqMan assay specific for ZIKV-RGN RNA. This TaqMan assay is constituted by the forward primer 5’-GAAGAGCATCCAGCCAGAGAA-3’ (spanning nucleotides 1358 to 1378 of the viral genome), the reverse primer 5’-CTGGGAGCCATGAACTGACA-3’ (complementary to nucleotides 1399 to 1418 of the viral genome), and the probe 5’-FAM-TGGAGTACCGGATAATG-3IABKFQ-3’ (covering nucleotides 1381 to 1397 of the viral genome). To normalize for differences in RNA sampling, the expression of the histone H2B (reference housekeeping gene) was analyzed using a specific TaqMan gene expression assay (Rh04253068_s1; Life Technologies, ThermoFisher Scientific). Data were acquired with a 7500 real-time PCR system (Life Technologies, ThermoFisher Scientific) and analyzed with ABI PRISM 7500 software v2.0.6. The relative quantifications were performed using the cycle threshold (2^−ΔΔCT^) method [42]. All experiments and data analysis were MIQE (Minimum Information for Publication of Quantitative Real-Time PCR Experiments) compliant [43].

### 2.9. Indirect Immunofluorescence Assay

The expression of ZIKV E protein was analyzed by indirect immunofluorescence assay (IFA). Vero cells grown on coverslips in 24-well plates at 80–90% of confluence were infected with the rescued rZIKVs at the indicated MOIs. At selected time points post-infection, cells were fixed with 4% paraformaldehyde in 250 mM Hepes pH 7.4 during 20 min at room temperature and then permeabilized with 0.5% Triton X-100 in PBS for 10 min. After that, cells were treated for 1 h at room temperature with blocking solution (10% FBS in PBS) and incubated with 1 µg/mL of the pan-flavivirus E protein mAb 4G2 (BEI Resources; NR-50327) in blocking solution for 2 h at room temperature. After three washed with PBS, cells were incubated at room temperature for 1 h with donkey anti-mouse antibody conjugated to Alexa Fluor 488 (Invitrogen, ThermoFisher Scientific) diluted 1:500 in blocking solution, extensively washed with PBS, and incubated for 10 min with DAPI (4’,6’-diamidino-2-phenylindole) (Sigma-Aldrich) diluted 1:200 in PBS for nuclear staining. Finally, coverslips were mounted in ProLong Gold antifade reagent (Invitrogen, ThermoFisher Scientific) and analyzed on a Leica SP5 confocal microscope. Immunofluorescence acquired images were processed and analyzed with ImageJ 1.52b software [44].

### 2.10. Enzyme-Linked Immunosorbent Assay

For the evaluation of the virus-specific antibodies levels present in the sera of vaccinated mice, enzyme-linked immunosorbent assays (ELISAs) were performed as previously described [45]. Briefly, 96-well plates were coated with cell lysates from mock- or ZIKV-infected Vero cells and incubated overnight at 4 °C. The coated wells were washed with PBS, blocked with 1% BSA in PBS, and then incubated with two-fold dilutions (starting dilution of 1:50) of mice sera for 1 h at 37 °C. After that, plates were washed with water and incubated with HRP-conjugated goat anti-mouse IgG (1:2000; Southern Biotech, Birmingham, AL, USA) for 1 h at 37 °C. Reactions were developed with tetramethylbenzidine (TMB) substrate (BioLegend, San Diego, CA, USA) for 10 min at room temperature, quenched with 2 N H_2_SO_4_, and read at 450 nm in a Vmax Kinetic microplate reader (Molecular devices, San Jose, CA, USA).

### 2.11. Mice Experiments

The in vivo studies were performed in type-I interferon (IFN) receptor deficient (IFNR-/-) A129 mice (The Jackson Laboratory, Bar Harbor, ME, USA) maintained in the animal care facility at the University of Rochester under specific pathogen-free conditions. In this animal model, subcutaneous (s.c.) or intraperitoneal (i.p.) infection with ZIKV induces neurological disease and the animals succumb to viral infection, with high viral load in blood, brain, spin cord, and testes, consistent with manifestations of ZIKV infection in humans. Although deficient in innate IFN responses, A129 mice retain their adaptive immunity and have been successfully used as a suitable model for testing antivirals and vaccines [46,47,48].

To evaluate virus pathogenicity, female 4-to-6-week-old A129 mice (*n* = 5) were first anesthetized i.p. with a mixture of ketamine (100 µg per gram of body weight) and xylazine (20 µg per gram of body weight), and then mock-infected (PBS) or infected s.c. in the footpad with the indicated doses of rZIKV-RGN or rZIKV-RGN-mNS2A diluted in PBS in a final volume of 50 µL. After viral infection, animals were monitored daily for morbidity (body weight loss and disease signs, including hunching, ruffling and hind limb paralysis) and mortality (survival) over 14 days. Mice showing more than 20% of body weight loss or severe paralysis were considered to have reached the experimental endpoint and were humanely euthanized. To correlate development of clinical symptoms and death with virus replication, 4-to-6-week-old mice (*n* = 6) were infected as described above and the viral titers in serum were determined at days 2 (*n* = 3) and 4 (*n* = 3) by plaque assay and immunostaining using the pan-flavivirus E protein mAb 4G2 as indicated before.

To evaluate the protection efficacy of the rZIKV-RGN-mNS2A, female 4-to-6-week-old A129 mice (*n* = 5) were first anesthetized i.p. as indicated above, and then mock-immunized (PBS) or immunized s.c. in the footpad with 10^5^ PFU of rZIKV-RGN-mNS2A diluted in PBS in a final volume of 50 µL. At 20 days post-immunization, mouse sera were collected by submandibular bleeding and the presence of total antibodies against ZIKV-RGN was evaluated by ELISA. Twenty-four hours after bleeding, mice were challenged s.c. in the footpad with 10^5^ PFU of rZIKV-RGN and their morbidity and mortality monitored over 14 days as previously described. To determine viral replication, challenged 4-to-6-week-old A129 mice (*n* = 6) were bleeding at days 2 (*n* = 3) and 4 (*n* = 3) post-challenge and ZIKV viremia was determined by plaque assay and immunostaining using the pan-flavivirus E protein mAb 4G2 as previously described.

### 2.12. Statistical Analysis

For quantitative analyses, a two-tailed, unpaired Student *t* test was used to analyze differences in mean values between groups. All results were expressed as mean ± standard deviations of the means. *P* values of <0.05 were considered significant. For mice experiments, the Meier Log-Rank test was used to compare survival data and the Reed and Muench method to determine the mouse lethal dose 50 (MLD_50_). GraphPad Prism v7.0 software was used for all statistical analysis.

### 2.13. Ethics Statement

All animal protocols were approved by the University of Rochester Committee of Animal Resources (Protocol number: UCAR-2017-005/101851; approval date: 05/05/2017) and complied with the recommendations in the Guide for the Care and Use of Laboratory animals of the National Research Council [49].

## 3. Results

### 3.1. Development of a ZIKV-RGN Reverse Genetic System Using a BAC

To overcome the toxicity problems associated to several flavivirus sequences during its propagation in bacteria, we used the BAC plasmid pBeloBAC11 (a single-copy plasmid derived from the *E. coli* F-factor) [40] to assemble a ZIKV infectious cDNA clone, based on the genome sequence of the RGN strain of ZIKV (GenBank accession number KU527068) [19] (Figure 1). This ZIKV-RGN strain was selected because it has no laboratory passage history and the full-length genome sequence was obtained from a ZIKV-infected fetus with microcephaly in 2015 [19], constituting a good candidate to further study ZIKV pathogenesis.

After appropriate selection of unique restriction sites in the ZIKV-RGN genome (Figure 1A), four overlapping DNA fragments (ZIKV 1 to ZIKV 4), spanning the full-length viral genome and flanked for the selected restriction sites, were chemically synthesized, and sequentially cloned into pBeloBAC11 to generate the infectious cDNA clone pBAC-ZIKV-RGN (Figure 1B). Fragment ZIKV 1 contained the CMV immediate-early promoter to allow the expression of the viral RNA in the nucleus by the cellular RNA polymerase II [50] and fragment ZIKV 4 was flanked at the 3’-end by the HDV ribozyme followed by the BGH termination and polyadenylation sequences to produce synthetic RNAs bearing authentic 3’-ends of the viral genome. This DNA-lunched system ensures capping of the viral RNA and allows the recovery of infectious virus from the transfected cDNA clone without the need of an in vitro transcription step. Once assembled, the full-length sequence of the ZIKV-RGN BAC clone was determined and no changes were detected to that reported for the ZIKV-RGN strain (GenBank accession number KU527068). Finally, to confirm the stability of this synthetic infectious cDNA clone in bacteria, the BAC clone was passaged in *E. coli* DH10B cells for more than two hundred generations and the genetic integrity of the passaged infectious clone analyzed by restriction endonuclease analysis and sequencing. No differences were detected, demonstrating that the ZIKV-RGN BAC clone was fully stable in bacteria and that the BAC approach is a reliable and simple method to generate ZIKV infectious cDNA clones.

### 3.2. Rescue and In Vitro Characterization of rZIKV-RGN

**Figure 2:**
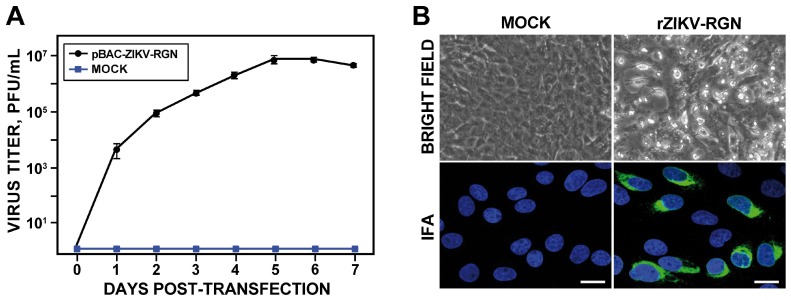
Recovery of infectious rZIKV-RGN from the BAC cDNA clone. (**A**) Virus rescue. Vero cells at 90% of confluence (6-well plate format; triplicates) were mock-transfected (Mock) or transfected with 4 µg/well of the ZIKV BAC cDNA clone (pBAC-ZIKV-RGN) and at the indicated times post-transfection, virus titers in the tissue culture supernatant of transfected cells were determined by plaque assay. Error bars represent standard deviations of the means from three experiments. (**B**) Analysis of the cytopathic effect (CPE) induction and ZIKV E protein expression. Vero cells were mock-infected (left) or infected (right) with 0.5 PFU/cell of the rescued virus (rZIKV-RGN) and at 48 h post-infection (hpi) analyzed for the induction of CPE by light microscopy (top) and for viral E protein expression by immunofluorescence assay (IFA) using the pan-flavivirus E protein mAb 4G2 (bottom). Bars, 20 µm.

To recover the infectious virus (Figure 2), Vero cells were transiently transfected with the BAC cDNA clone using Lipofectamine 2000 and virus production analyzed during seven days. In contrast to mock-transfected cells, increasing amounts of infectious virus were detected in the tissue culture supernatant of cells transfected with the infectious clone, with peak titers around 10^7^ PFU/mL on day five (Figure 2A). To further confirm the identity of the rescued virus, Vero cells were infected with an MOI of 0.5 PFU/cell of the rescue virus and monitored for CPE induction and viral E protein expression by IFA using the pan-flavivirus E protein mAb 4G2 (Figure 2B). The rescued virus induced a clear CPE, characterized by the presence of rounded and birefringent cells, and high levels of E protein expression were detected in the perinuclear region of infected cells. These results demonstrated that the ZIKV-RGN infectious BAC cDNA clone produces high titers of rZIKV-RGN directly after transfection of susceptible Vero cells.

**Figure 3:**
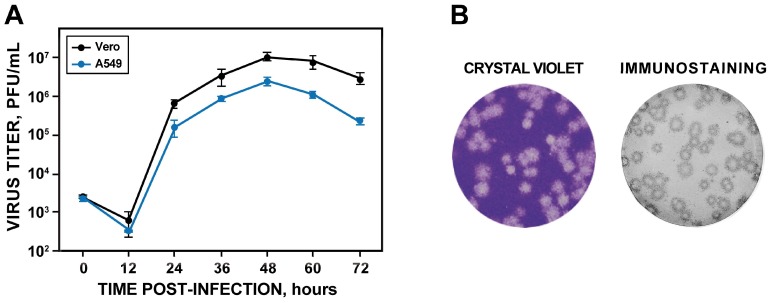
Viral growth kinetics and plaque phenotype of rZIKV-RGN. (**A**) Growth kinetics. Vero and A549 cells at 90% confluence (24-well plate format; triplicates) were infected at a multiplicity of infection (MOI) of 1 PFU/cell and at the indicated hpi, virus titers in the tissue culture supernatants were determined by plaque assay on Vero cells. Error bars represent standard deviations of the mean from three experiments. (**B**) Plaque morphology. Vero cells at 90% confluence (6-well plate format) were infected with 50 PFU of rZIKV-RGN and at four days post-infection viral plaques were visualized by staining with crystal violet (left) or immunostaining (right) using the pan-flavivirus E protein mAb 4G2.

Once the identity of the rescued virus was confirmed, it was cloned by three round of plaque purification, and its phenotypic and genotypic properties were determined. Analysis of the growth kinetics revealed that the rZIKV-RGN replicated efficiently in both Vero and A549 cells, reaching peak titers of approximately 10^7^ and 10^6^ PFU/mL at 48 hpi, respectively (Figure 3A). In addition, the rescued virus generated homogeneous plaques of about 2 mm in size after four days of infection in Vero cells (Figure 3B). Finally, the genetic identity of the virus was analyzed by deep-sequencing of two independent clones. Full-genome sequencing of both viral clones revealed that both clones presented the same sequence that the cDNA clone. Overall, these results demonstrate the feasibility of generating infectious rZIKVs using a BAC-based approach.

### 3.3. Pathogenesis of rZIKV-RGN in Mice

**Figure 4:**
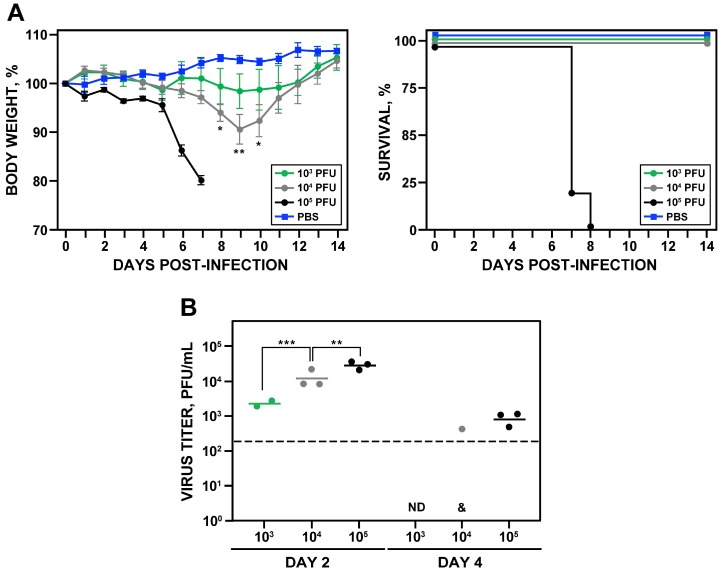
Pathogenesis of rZIKV-RGN in A129 mice. (**A**) Weight loss and mortality. Female 4-to-6-week-old A129 mice (five mice per group) were mock-infected (PBS) or infected s.c. in the footpad with the indicated PFU of rZIKV-RGN, and body weight loss (expressed as the percentage of starting weight, left panel) and survival (right panel) were monitored daily during 14 days. Mice that lost more than 20% of their initial body weight or presented hind limb paralysis were humanely euthanized. Error bars represent standard deviations of the mean for each group of mice. Asterisks indicate that the differences between viral doses of 10^3^ and 10^4^ are statistically significant when data are compared using the unpaired *t* test (*, *P* < 0.05; **, *P* < 0.01). (**B**) Viral titers in mice sera. Female 4-to-6-week-old A129 mice (six mice per group) were infected with the indicated PFU of rZIKV-RGN as described above, and viral titers in sera were determined at days two and four after infection (three animals per time point) by plaque assay and immunostaining using the pan-flavivirus E protein mAb 4G2. Symbols represent data from individual mice and bars the geometric means of viral titers. Asterisks indicate that the differences in viral titers between experimental samples are statistically significant when data are compared using the unpaired *t* test (**, *P* < 0.01; ***, *P* < 0.001). &: virus not detected in two mice; ND: virus not detected. The detection limit of the assay (200 PFU/mL) is indicate as a dashed line.

To determine whether the rescued rZIKV-RGN was pathogenic in vivo (Figure 4), groups of five female 4-to-6-week-old A129 mice (IFNR-/-) were inoculated s.c. in the footpad with PBS (as negative control) or with different doses of rZIKV-RGN (10^3^, 10^4^ and 10^5^ PFU per animal) and the morbidity (body weight loss and disease signs) and survival were monitored daily over 14 days (Figure 4A). As expected, weight loss and survival correlated with the inoculated dose. Mice infected with 10^3^ PFU did not show disease symptoms, only a slight reduction in body weight was detected on days 8 to 12, and all of them survived. In the case of mice infected with 10^4^ PFU, they presented some symptoms of disease (hunching and reduced mobility) and weight loss from days seven to nine (with a maximum of 10% on day nine), but all of them recovered the initial body weight and survived. In contrast, animals infected with 10^5^ PFU showed hind limb paralysis, rapidly lost weight, and all of them succumbed to viral infection between days seven and eight post-infection (Figure 4A). Using the Reed & Muench method we determined that the MLD_50_ of rZIKV-RGN was approximately 5 × 10^4^ PFU. To further analyze whether the virulence observed correlated with viral replication, viral titers in mouse sera were analyzed at days two and four post-infection (Figure 4B). As expected, the viremia in the infected animals was dose dependent, reaching the highest titers at day two after inoculation. At day four post-inoculation a significant reduction of the viral titers was observed. In the case of animals infected with 10^3^ or 10^4^ PFU, viremia was not detected or only detected in one of the three infected mice, respectively (Figure 4B). Overall, these results indicated that the rZIKV-RGN recovered from the infectious clone is virulent in mice but only at high (10^5^ PFU) dose.

### 3.4. Rescue and In Vitro Charazterization of a rZIKV-RGN Harboring a Point Mutation in the NS2A Protein

**Figure 5:**
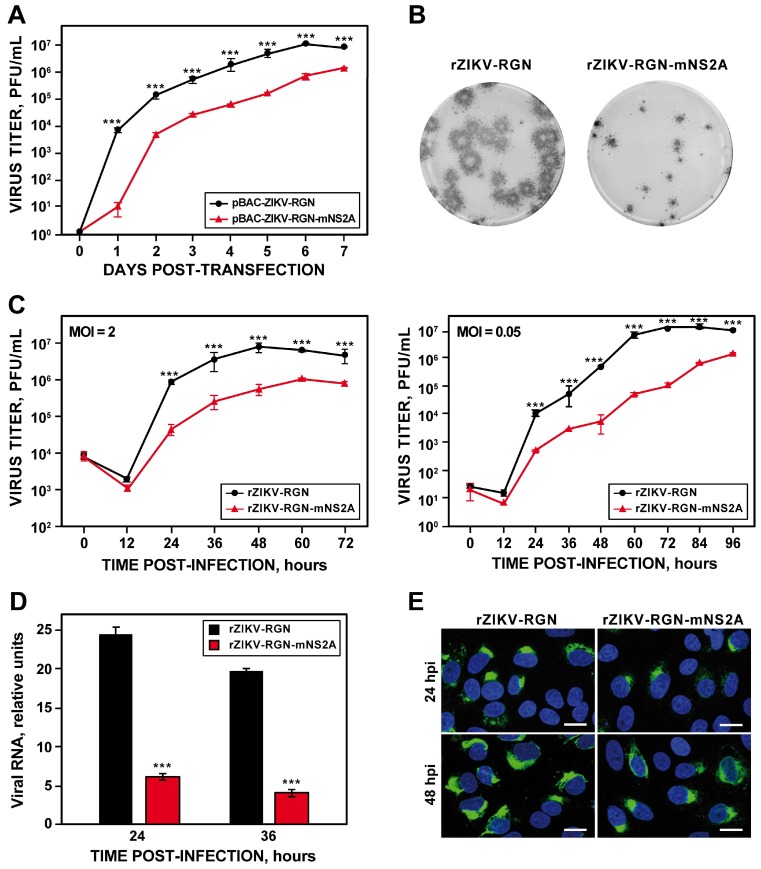
Rescue and growth properties of rZIKV-RGN-mNS2A in Vero cells. (**A**) Virus rescue. Vero cells at 90% of confluence (6-well plate format; triplicates) were transiently transfected with 4 µg/well of the infectious clones pBAC-ZIKV-RGN or pBAC-ZIKV-RGN-mNS2A, and at the indicated times post-transfection, virus titers in tissue culture supernatants were determined by plaque assay. Error bars represent standard deviations of the means from three experiments. (**B**) Plaque morphology. Vero cells at 90% confluence (6-well plate format) were infected with 25 PFU of rZIKV-RGN or rZIKV-RGN-mNS2A and at four days post-infection the viral plaques were visualized by immunostaining using the pan-flavivirus E protein mAb 4G2. (**C**) Growth kinetics. Vero cells at 90% confluence (24-well plate format; triplicates) were infected with rZIKV-RGN or rZIKV-RGN-mNS2A at high (2 PFU/cell, left) or low (0.05 PFU/cell, right) MOI, and at the indicated hpi virus titers were determined by plaque assay. Error bars represent standard deviations of the mean from three experiments. (**D**) Analysis of viral RNA synthesis. Vero cells at 90% confluence (12-well plate format; triplicates) were infected (MOI of 0.5 PFU/cell) with rZIKV-RGN or rZIKV-RGN-mNS2A and at the indicated hpi, viral RNA levels were quantified by RT-qPCR. Error bars represent standard deviations of the mean from three experiments. (**E**) Analysis of viral E protein expression. Vero cells were infected (MOI of 0.5 PFU/cell) with rZIKV-RGN or rZIKV-RGN-mNS2A and at the indicated hpi, viral E protein expression was analyzed by IFA using the pan-flavivirus E protein mAb 4G2. Bars, 20 µm. Asterisks in panels A, C, and D indicate that the differences between rZIKV-RGN and rZIKV-RGN-mNS2A are statistically significant when data are compared using the unpaired *t* test (***, *P* < 0.001).

During the assembly of the pBAC-ZIKV-RGN infectious clone, we detected the presence of a point mutation in the NS2A protein, which was introduced during the chemical synthesis of fragment ZIKV 2. This mutation consists in a cytosine-to-thymidine substitution at genomic position 4069, resulting in an alanine-to-valine change in the residue 175 of the NS2A protein (A175V). Because this mutation consists of a conservative amino acid change, we decided to explore the possibility of using this mutation as a genetic marker. To this end, the infectious clone pBAC-ZIKV-RGN-mNS2A was generated by replacing the ZIKV 2 WT fragment for that containing the NS2A A175V mutation. This infectious clone was fully stable in bacteria and no additional mutations were observed after sequencing the full-length clone. After that, Vero cells were transfected with the mutant infectious clone and the recovery efficiency of the rZIKV-RGN-mNS2A mutant virus was compared to that of the parental rZIKV-RGN virus (Figure 5). Although the infectious virus was recovered in both cases, virus production was one logarithm lower in the case of the mutant rZIKV-RGN-mNS2A, reaching maximum titers of 10^6^ PFU/mL at seven days post-transfection (Figure 5A). When the plaque phenotype was analyzed, we found that the plaque size of the mutant rZIKV-RGN-mNS2A was smaller (more than a 5-fold reduction) than that of the parental rZIKV-RGN (Figure 5B), indicating that the A175V mutation, despite of being a conservative substitution, caused reduction in plaque size and virus production. In addition, an in silico analysis was performed to evaluate the frequency of amino acid residues 175 of the NS2A protein in more than 700 ZIKV strains sequences deposited in the database [51] (https://www.viprbrc.org/brc/home.spg?decorator=flavi). This analysis indicated that amino acid A175 is highly conserved, since the 100% of the analyzed ZIKV sequences contained an alanine residue at this position.

To further confirm the effect of the NS2A A175V mutation on virus production, the growth kinetics at high (2 PFU/cell) and low (0.05 PFU/cell) MOI of the mutant virus were compared to those of the parental virus (Figure 5C). Again, a reduction of about one logarithmic unit in virus production was detected in Vero cells infected with the mutant virus both at high and low MOI (Figure 5C). Taken into consideration that flavivirus NS2A protein is involved in regulation of RNA replication and virus assembly [5], we further analyzed whether the reduction in plaque size and virus production of the mutant virus was associated with reduced viral RNA synthesis. To this end, the production of viral RNA in Vero cells infected with either the parental or mutant viruses at an MOI of 0.5 PFU/cell was analyzed at 24 and 36 hpi by RT-qPCR using a custom TaqMan assay specific for ZIKV-RGN genome (Figure 5D). At both times, a 5-fold reduction in the levels of viral RNA was observed in cells infected with the mutant virus (Figure 5D), confirming that NS2A A175V mutation at least impairs viral RNA synthesis. In agreement with these data, a reduction in the expression levels of ZIKV E protein was observed by IFA in Vero cells infected with the mutant virus in comparison to cells infected with the parental virus (Figure 5E). Finally, to discard the presence of other undesired mutations, the full-length sequence of the mutant virus was determined by deep-sequencing, and no mutations other than NS2A A175V were detected. Collectively, these results indicated that NS2A A175V mutation alone affected ZIKV growth in Vero cells at least by impairing viral RNA synthesis.

### 3.5. Pathogenesis of rZIKV-RGN-mNS2A in Mice

**Figure 6:**
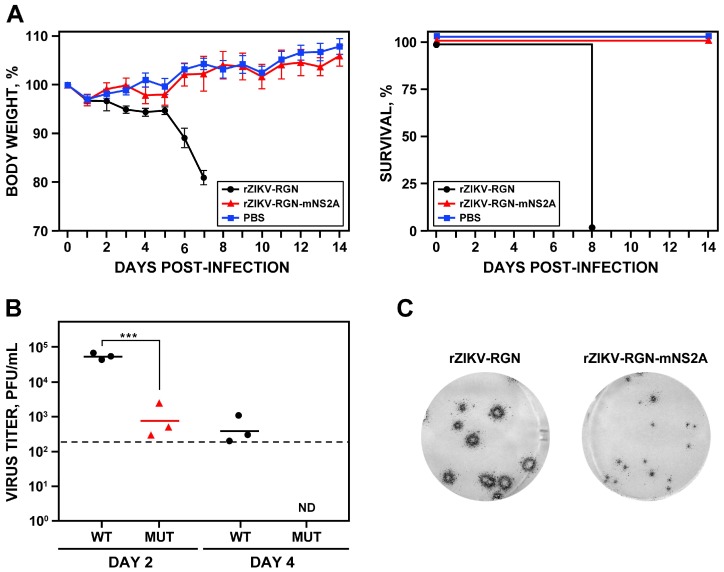
Pathogenesis of rZIKV-RGN-mNS2A in A129 mice. (**A**) Weight loss and mortality. Female 4-to-6-week-old A129 mice (five mice per group) were mock-infected (PBS) or infected s.c. in the footpad with 10^5^ PFU of rZIKV-RGN or rZIKV-RGN-mNS2A, and body weight loss (expressed as the percentage of starting weight, left panel) and survival (right panel) were monitored daily during 14 days. Mice that lost more than 20% of their initial body weight or presented hind limb paralysis were humanely euthanized. Error bars represent standard deviations of the mean for each group of mice. (**B**) Viral titers in mice sera. Female 4-to-6-week-old A129 mice (six mice per group) were infected with 10^5^ PFU of rZIKV-RGN (WT) or rZIKV-RGN-mNS2A (MUT) as described above, and viral titers in sera were determined at days two and four after infection (three animals per time point) by plaque assay and immunostaining using the pan-flavivirus E protein mAb 4G2. Symbols represent data from individual mice and bars the geometric means of viral titers. Asterisks indicate that the differences between rZIKV-RGN and rZIKV-RGN-mNS2A are statistically significant when data are compared using the unpaired *t* test (***, *P* < 0.001). ND: virus not detected. The detection limit of the assay (200 PFU/mL) is indicate as a dashed line. (**C**) Plaque phenotype. Vero cells at 90% confluence (6-well plate format) were infected with 25 PFU of rZIKV-RGN (left) or rZIKV-RGN-mNS2A (right) recovered from infected mice at day two post-infection and the plaque size evaluated by plaque assay and immunostaining using the pan-flavivirus E protein mAb 4G2.

To investigate whether the reduced RNA synthesis of rZIKV-RGN-mNS2A in Vero cells could result in viral attenuation in vivo, the ability of the mutant virus to induce pathogenesis was analyzed in A129 mice and compared with that of the parental rZIKV-RGN (Figure 6). To that end, groups of five female 4-to-6-week-old A129 mice were inoculated s.c. in the footpad with 10^5^ PFU of either rZIKV-RGN or rZIKV-RGN-mNS2A, or with PBS as a negative control, and the body weight loss and survival were monitored daily over 14 days. In contrast to mice infected with rZIKV-RGN that quickly lost weight and all of them died at day eight after infection, mice infected with the mutant rZIKV-RGN-mNS2A did not presented any clinical signs of infection or weight loss and all of them survived to viral infection (Figure 6A). To further analyze the correlation of the attenuation of the mutant virus with viral replication, presence of the virus in mice sera was analyzed at days two and four post-infection. In agreement with the pathogenicity data, mice infected with the mutant virus presented lower viremia than mice infected with the parental virus (Figure 6B). The mutant virus was only detected at day two after infection and at lower titers (approximately 1.5 logarithms lower) than the parental virus. As an internal control of the experiment, the plaque phenotype of the viruses recovered from the blood of infected mice were analyzed. As expected, rZIKV-RGN formed big plaques while the mutant rZIKV-RGN-mNS2A formed small plaques (Figure 6C). These results indicated that rZIKV-RGN-mNS2A was highly attenuated in mice, as compared to rZIKV-RGN, and that this attenuation may be due to a lower replication of the rZIKV-RGN-mNS2A mutant virus.

### 3.6. Analysis of the Protection Efficacy in Mice of rZIKV-RGN-mNS2A

**Figure 7:**
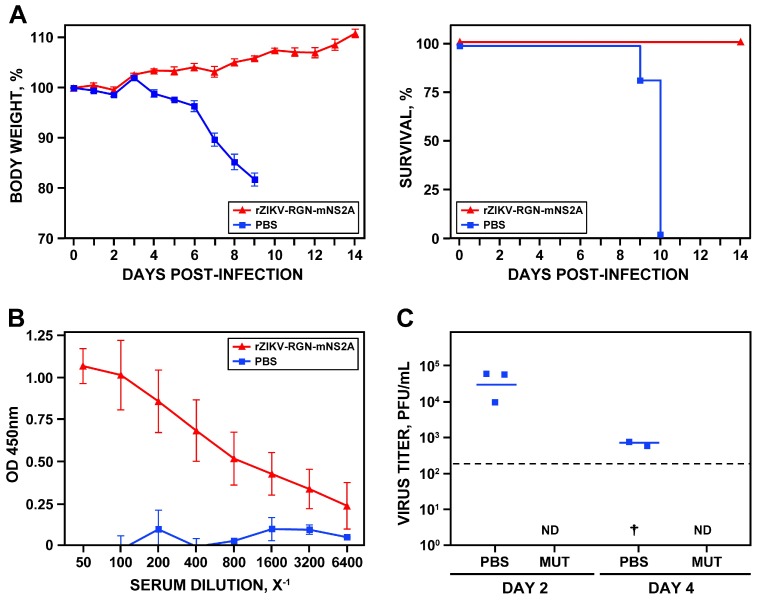
Protection efficacy of rZIKV-RGN-mNS2A. (**A**) Weight loss and mortality. Female 4-to-6-week-old A129 mice (five mice per group) were mock-vaccinated (PBS) or vaccinated s.c. in the footpad with 10^5^ PFU of rZIKV-RGN-mNS2A. At 21 days after vaccination, mice were challenged with 10^5^ PFU of rZIKV-RGN and the body weight loss (expressed as the percentage of starting weight, left panel) and survival (right panel) were monitored daily during 14 days. Mice that lost more than 20% of their initial body weight or presented hind limb paralysis were humanely euthanized. Error bars represent standard deviations of the mean for each group of mice. (**B**) Induction of humoral response. One day before challenge with rZIKV-RGN, sera samples were collected from mock-vaccinated (PBS) and rZIKV-RGN-mNS2A vaccinated mice, and total IgG antibodies against ZIKV-RGN were evaluated by ELISA. OD, optical density. Error bars represent standard deviations of the mean for each group of mice. (**C**) Viral titers in mice sera. Female 4-to-6-week-old A129 mice (six mice per group) mock-vaccinated (PBS) or vaccinated with 10^5^ PFU of rZIKV-RGN-mNS2A (MUT) were challenged with 10^5^ PFU of rZIKV-RGN as described above, and viral titers in sera were determined at days two and four after challenge (three animals per time point) by plaque assay and immunostaining using the pan-flavivirus E protein mAb 4G2. Symbols represent data from individual mice and bars the geometric means of viral titers. †: virus not detected in one mouse; ND: virus not detected. The limit of detection of the assay (200 PFU/mL) is indicate as a dashed line.

After elucidating that rZIKV-RGN-mNS2A was attenuated in vivo, its ability to induce protection against a challenge with the parental rZIKV-RGN was analyzed (Figure 7). To that end, groups of five female 4-to-6-week-old A129 mice were vaccinated s.c. in the footpad with 10^5^ PFU of rZIKV-RGN-mNS2A or mock-vaccinated with PBS. Twenty days after vaccination, blood samples were collected to evaluate the humoral response. One day later, mice were challenged with a lethal dose (10^5^ PFU) of rZIKV-RGN and the body weight loss and survival were analyzed daily over 14 days. As expected, mice vaccinated with PBS lost weight rapidly, showed clear symptoms of disease, and all of them succumbed to challenge with rZIKV-RGN. In contrast, mice vaccinated with rZIKV-RGN-mNS2A did not lose weight and all of them survived the challenge with rZIKV-RGN (Figure 7A), indicating that a single immunization dose with rZIKV-RGN-mNS2A is enough to induce full protection against ZIKV-RGN. In agreement with these data, a strong humoral response against ZIKV-RGN was observed in mice vaccinated with rZIKV-RGN-mNS2A (Figure 7B) and no viremia was detected in sera samples of vaccinated mice at days two and four after challenge (Figure 7C).

### 3.7. Genetic Stability of rZIKV-RGN-mNS2A In Vero Cells

**Figure 8:**
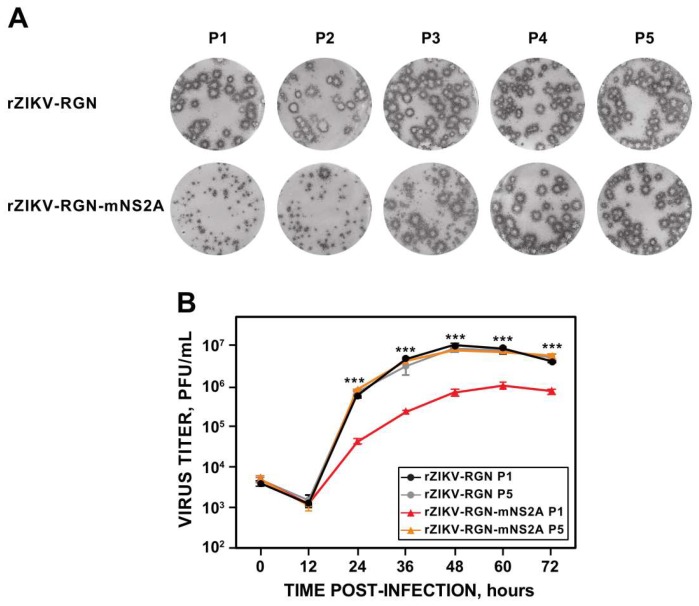
Stability of rZIKV-RGN-mNS2A in cultured cells. Vero cells growth in 6-well plates at 90% of confluence were infected with 0.1 PFU/cell of rZIKV-RGN or rZIKV-RGN-mNS2A. At 72 hpi, cell culture supernatants were collected and used to infect fresh Vero cells. This process was repeated four more times and virus stocks of passages 1 to 5 (P1 to P5) were generated. (**A**) Plaque size. Vero cells were infected with the different passages (P1 to P5) of rZIKV-RGN or rZIKV-RGN-mNS2A, and at four days post-infection the viral plaques were visualized by immunostaining using the pan-flavivirus E protein mAb 4G2. (**B**) Growth kinetics. Vero cells at 90% confluence (24-well plate format; triplicates) were infected (MOI of 1 PFU/cell) with P1 and P5 of rZIKV-RGN or rZIKV-RGN-mNS2A and at the indicated hpi, virus titers were determined by plaque assay. Error bars represent standard deviations of the mean from three experiments. Asterisks indicate that the differences between rZIKV-RGN-mNS2A P1 and the experimental samples, rZIKV-RGN P1, rZIKV-RGN P5 and rZIKV-RGN-mNS2A P5, are statistically significant when data are compared using the unpaired *t* test (***, *P* < 0.001).

Once confirmed that rZIKV-RGN-mNS2A was attenuated in vivo and induces protection against ZIKV-RGN in mice, the genetic stability of the mutant virus was analyzed in Vero cells, in order to test the possible use of this mutant virus as a base for the development of a live-attenuated ZIKV vaccine (Figure 8). To this end, both mutant (rZIKV-RGN-mNS2A) and parental (rZIKV-RGN) viruses were passaged five times in Vero cells (P1 to P5) and the virus plaque phenotype, growth kinetics and the sequence of NS2A were analyzed for each passage.

Analysis of the plaque phenotype showed that the parental virus presented the expected plaque size throughout all passaging. In contrast, for the mutant virus a reversion to parental plaque phenotype was observed. This reversion started at P2 (2% of big plaques), clearly increased at P3 (45% of big plaques) and was complete at P4 (Figure 8A). After that, we analyzed whether this plaque size reversion of the mutant rZIKV-RGN-mNS2A correlated with an increase in virus replication to levels of that of the parental virus. To that end, the growth kinetics (MOI of 1 PFU/cell) of the mutant virus from P1 and P5 were compared to those of the parental virus. Growth curve analysis showed that in contrast to the mutant virus from P1, the virus from P5 replicated to the same levels as the parental virus (Figure 8B), suggesting that the mutant virus reverted to the WT sequence during its propagation in Vero cells. To confirm these observations, the NS2A coding region of mutant viruses from P1 to P5 was amplified by RT-PCR and sequenced. Sequence analysis confirmed the reversion of the A175V mutation to the WT sequence. Although the instability and reversion of the mutant virus to the WT sequence during its propagation in Vero cells limits the use of this mutant for vaccine development, these data further support the importance of this NS2A residue for virus replication. Moreover, these data also suggest that ZIKV NS2A protein represents a good target for the development of antivirals against ZIKV infection.

## 4. Discussion

The significance of ZIKV to public health due its association with Guillain–Barré syndrome and fetal abnormalities [14,15,16,17,18,19], together with the lack of approved antiviral agents or vaccines, have triggered a global effort to study this flavivirus in order to develop effective strategies to prevent and control ZIKV infection in humans. In this respect, the development and implementation of reverse genetic approaches for ZIKV provide investigators with a novel and powerful experimental tool to study both the biology and pathogenesis of ZIKV as well as the development of attenuated forms of ZIKV for their implementation as live-attenuated vaccines. However, as described for other flaviviruses, the generation of ZIKV infectious clones using traditional approaches are very difficult due to the toxicity and instability of some viral sequences when they were propagated as cloned cDNA in bacteria [24,25,26]. In the past two years, several approaches that overcomes this toxicity problem have been applied for the successfully generation of ZIKV infectious clones. These include the use of low-copy plasmids [29,30], insertion of introns to disrupt toxic sequences [31,32,33], mutational silencing of CBPs present in the viral genome [37], in vitro ligation of cDNA fragments [27,28,34], the ISA method [35,36], and the CPER approach [38]. Although very useful, some of these approaches are laborious, time consuming and present several disadvantages. For instance, most of them need in vitro ligation and transcription steps that complicate the assembly and reduce the recovery efficiency. Moreover, these low recovery efficiencies increase the presence of undesired mutations that could result in in vitro and/or in vivo attenuation, limiting the use of these infectious clones for certain studies. Others, such as the mutational silencing of CBPs, in which a high number of silent mutations have to be introduced, could affect viral fitness. Finally, the use of low-copy plasmids has been shown to be effective for several ZIKV strains but not for others, probably due the different degrees of toxicity of RNA sequences of different strains [28,32,34,36].

Here, we describe a powerful approach for the generation of an infectious cDNA clone of the ZIKV-RGN strain in a single plasmid, based on the use of a combination of synthetic biology and BACs. The full-length cDNA copy of the ZIKV-RGN strain was generated from four synthetic DNA fragments and cloned in the BAC plasmid pBeloBAC11 [40] under the control of the CMV promoter, which allows the expression of the viral RNA in the nucleus [50], and flanked at the 3’-end by the HDV ribozyme and the BGH polyadenylation and termination sequences to produce synthetic RNAs bearing authentic 3’-ends of the viral genome. The BAC cDNA clone was fully stable during its propagation in bacteria and the functional infectious virus was rescued after direct transfection of susceptible Vero cells that was pathogenic in A129 mice. A ZIKV-RGN infectious clone generated using the CPER approach has been recently reported [38]. However, in contrast to our results, the rescued virus was asymptomatic and nonlethal in female 8-to-12-week-old A129 mice infected with doses of 10^3^ to 10^6^ CCID_50_ (50% cell culture infective doses) via the s.c. route. Whether the differences in pathogenicity among this rZIKV-RGN and ours are related to the experimental approach (CPER versus BAC), the age of the mice (8-to-12-week-old versus 4-to-6-week-old) or the MOI used to infect the mice (10^3^–10^6^ CCID_50_ versus 10^5^ PFU) remain to be evaluated.

Although other ZIKV reverse genetic systems have been reported (discussed above), the BAC approach constitutes an useful alternative that presents important advantages: (i) The BAC plasmids present a strictly controlled replication leading to only one plasmid per cell and therefore minimize the toxicity associated with several flavivirus sequences when amplified in bacteria [41]. This allows the easy and direct manipulation of the viral genome for molecular studies; (ii) Similarly to other approaches using polII-driven promoters, the BAC approach results in intracellular expression of the viral RNA [32,33,35,36,38], allowing the capping of the viral RNA and the recovery of infectious virus without the need of an in vitro transcription step. Although some splicing events could occur during the nuclear expression of the viral genome, mainly due to the presence of donor and acceptor putative sequences in the viral genome, the efficiency of this phenomenon is very low and does not affect the recovery of infectious viruses [52]; (iii) Like other systems based on transfection of DNA constructs [32,33,35,36,38], BAC cDNA clones present a higher efficiency of transfection than RNA transcripts in mammalian cells. This allows higher efficiencies of virus recovery, reducing the passages in cell culture to get a viral stock and therefore, the possibility of introducing undesired mutations by cell culture adaptation; (iv) The manipulation of BAC cDNA clones is relatively easy and similar to that of conventional plasmids with slight modifications due to the presence of only one plasmid copy per cell. In addition to standard protocols, the BAC cDNA clones could also be efficiently modified into *E. coli* by homologous recombination using a two-step approach that combine the Red recombination system and counterselection with the homing endonuclease I-SceI [53,54,55,56]; and (v) The BAC approach has been successfully used to engineer infectious clones of other flaviviruses, including DENV [39], and several coronaviruses that contain the largest viral RNA genome known and similar toxicity problems to those described for flaviviruses [52,57,58,59,60]. These data highlight the potential of the BAC approach for the rapid and reliable construction of stable infectious clones of emerging flavivirus and other similar RNA viruses with unstable viral genomes when amplified as cDNAs in bacteria.

The ZIKV reverse genetic system described in this article was further used to study the effect of a single amino acid substitution (A175V) in the viral NS2A protein on virus growth in cultured cells and pathogenesis in vivo. Our results suggested that this single amino acid change impaired viral RNA synthesis and virus production in cell culture and highly attenuated the virus in mice. However, we cannot discard that this mutation in the NS2A protein could affect other steps in the replication cycle of the virus. Flavivirus NS2A protein is a 22-kDa hydrophobic protein associated with the endoplasmic reticulum that contains eight transmembrane domains. It is a multifunctional protein that has been involved in viral RNA synthesis [61,62], virus assembly [63,64], membrane rearrangement [64], and immunomodulation of innate immune response [65,66,67]. By homology with the DENV NS2A topology, the ZIKV NS2A A175V mutation maps in the last transmembrane domain, for which no specific function has been reported. Therefore, our data constitute the first evidence of a role of this NS2A domain in viral RNA synthesis. On the other hand, it is important to note that the NS2A A175V mutation promoted a 5-fold reduction in viral RNA synthesis and more than 10-fold reduction in virus production. This reduced virus production could be a consequence of the RNA synthesis impairment. However, since the reduction in virus production is higher than that observed in RNA synthesis and that flavivirus NS2A protein is also involved in virus assembly, we cannot discard an additional effect of A175V mutation in ZIKV assembly. In addition, we have found that the mutant virus was attenuated in A129 mice. This attenuation could be explained as a consequence of the lower RNA synthesis of the mutant virus. However, an additional effect of the A175V mutation on the putative immunomodulatory role of NS2A protein [65,66,67], leading to virulence attenuation, cannot be discarded. Future studies will be required to determine whether this mutation affects only viral RNA synthesis or also virus assembly and immunomodulation of the host defenses.

Importantly, we have shown that immunization with a single dose of 10^5^ PFU of the mutant rZIKV-RGN-mNS2A induced protection against a lethal challenge with the parental rZIKV-RGN, suggesting the potential implementation of this NS2A mutant as the base of a live-attenuated vaccine. Unfortunately, the mutant rZIKV-RGN-mNS2A was instable and reverted to the WT sequence during its propagation in Vero cells, limiting the use of this mutation alone for vaccine development. However, this instability and the high conservation of the amino acid A175 of the NS2A protein among ZIKV strains highlights the importance of this NS2A residue for virus replication, and therefore the potential use of NS2A protein as a good target for antiviral development against ZIKV infection.

In summary, we have developed a powerful ZIKV reverse genetic system based on the use of BACs that has allowed us to identify a single point mutation in the NS2A protein that attenuates the virus in vitro and in vivo. This infectious clone system provides a valuable tool to the research community to explore ZIKV molecular biology, viral determinants of ZIKV pathogenesis, virus-host interactions, and vaccine and antivirals developments.
